# ZINQ-L: a zero-inflated quantile approach for differential abundance analysis of longitudinal microbiome data

**DOI:** 10.3389/fgene.2024.1494401

**Published:** 2025-01-29

**Authors:** Shuai Li, Runzhe Li, John R. Lee, Ni Zhao, Wodan Ling

**Affiliations:** ^1^ Department of Biostatistics, Johns Hopkins Bloomberg School of Public Health, Baltimore, MD, United States; ^2^ Division of Nephrology and Hypertension, Department of Medicine, Weill Medical College of Cornell University, New York, NY, United States; ^3^ Department of Transplantation Medicine, New York Presbyterian Hospital–Weill Cornell Medical Center, New York, NY, United States; ^4^ Division of Biostatistics, Department of Population Health Sciences, Weill Medical College of Cornell University, New York, NY, United States

**Keywords:** longitudinal microbiome studies, zero inflation and disperson, mixed-effects models, quantile rank-score test, heterogeneous associations

## Abstract

**Background:**

Identifying bacterial taxa associated with disease phenotypes or clinical treatments over time is critical for understanding the underlying biological mechanism. Association testing for microbiome data is already challenging due to its complex distribution that involves sparsity, over-dispersion, heavy tails, etc. The longitudinal nature of the data adds another layer of complexity - one needs to account for the within-subject correlations to avoid biased results. Existing longitudinal differential abundance approaches usually depend on strong parametric assumptions, such as zero-inflated normal or negative binomial. However, the complex microbiome data frequently violate these distributional assumptions, leading to inflated false discovery rates. In addition, the existing methods are mostly mean-based, unable to identify heterogeneous associations such as tail events or subgroup effects, which could be important biomedical signals.

**Methods:**

We propose a zero-inflated quantile approach for longitudinal (ZINQ-L) microbiome differential abundance test. A mixed-effects quantile rank-score-based test was proposed for hypothesis testing, which consists of a test in mixed-effects logistic model for the presence-absence status of the investigated taxon, and a series of mixed-effects quantile rank-score tests adjusted for zero inflation given its presence. As a regression method with minimal distributional assumptions, it is robust to the complex microbiome data, controlling false discovery rate, and is flexible to adjust for important covariates. Its comprehensive examination of the abundance distribution enables the identification of heterogeneous associations, improving the testing power.

**Results:**

Extensive simulation studies and an application to a real kidney transplant microbiome study demonstrate the improved power of ZINQ-L in detecting true signals while controlling false discovery rates.

**Conclusion:**

ZINQ-L is a zero-inflated quantile-based approach for detecting individual taxa associated with outcomes or exposures in longitudinal microbiome studies, providing a robust and powerful option to improve and complement the existing methods in the field.

## 1 Introduction

The human microbiome plays a pivotal role in numerous diseases and health conditions, including diabetes (Qin et al., 2012), inflammatory bowel disease (Simrén et al., 2013), and HIV infections (Nowak et al., 2015). A central focus of microbiome research is the identification of specific taxa whose abundances significantly vary across different groups or conditions. Differential Abundance (DA) analysis offers crucial insights into the intricate interactions between microbial communities, their hosts, and their environments. This analysis enables researchers to discover microbial signatures linked to health and disease states, assess the effects of treatments or interventions, and accelerate the identification of potential therapeutic targets.

In past decades, various methods (Ritchie et al., 2015; Robinson et al., 2010; Love et al., 2014; Martin et al., 2020; Paulson et al., 2013) have been developed for differential abundance (DA) analysis, predominantly tailored for cross-sectional studies. In recent years, longitudinal study designs, which involve collecting repeated samples from the same subjects over time, are increasingly employed in microbiome research. Investigators benefit from the longitudinal studies as they facilitate the investigation of temporal dynamics of microbial communities, elucidate the forces that shape and sustain the microbiome, and thus enable the development and evaluation of microbiota-based interventions.

However, DA analysis of longitudinal microbiome data is particularly challenging due to its unique characteristics. The difficulties inherent to cross-sectional DA methods are also common in longitudinal studies. For instance, microbiome data is often sparse with considerable zeros in the Operational Taxonomic Unit (OTU) table. Additionally, the distribution of microbiome data tends to be heavy-tailed or skewed, which complicates modeling assuming parametric distributions. These challenges persist in longitudinal studies. Beyond these challenges, longitudinal designs introduce additional complexity that most DA methods, which do not account for the within-subject correlation structure, fail to address. While numerous DA approaches exist for cross-sectional microbiome data, there is a relative lack of methods accommodating longitudinal designs. In a recent benchmark study, Yang and Chen (2023) summarized the existing methods for correlated microbiome data into three broad categories. The first category employs classic linear mixed-effects models (LMM) on transformed data, with transformations including log transformation, centered log-ratio transformation (CLR), arcsine-square root transformation, etc. For instance, the default configuration of Microbiome Multivariable Association with Linear Models (MaAslin2) (Mallick et al., 2021) fits an LMM on the relative abundance data, and Linear models for Differential Abundance analysis (LinDA) (Zhou et al., 2022) applies an LMM to centered log-ratio-transformed data. The second category directly models the microbial relative abundance using parametric distributions supported on the interval [0,1], such as the beta distribution. An example is the two-part zero-inflated beta mixed-effects model (ZIBR) (Chen and Li, 2016), which fits a mixed-effects logistic model for the zero component and a mixed-effects beta regression model for the nonzero component. The third category encompasses a range of methods that model the taxonomic count via generalized linear mixed-effects models (GLMM). For example, Zhang and Yi (2020) proposed a series of GLMM-based approaches. One of their methods employs the negative binomial mixed-effects model (NBMM) to model the OTU counts, and another method extends it to the zero-inflated negative binomial mixed-effects model (ZINBMM) to accommodate the inflated zeros. Most of the methods, however, have limitations. First, they rely on specific probabilistic distributions, such as the negative binomial or Poisson distributions, which can be easily violated by the complexity of real microbiome data. Second, these methods typically identify the mean shift of taxa w.r.t. the clinical variable of interest, potentially overlooking heterogeneous associations arising from differences in distribution. For instance, even if the overall average is similar, differences in distributions could stem from tail events or subgroup effects that these methods may fail to detect. Thirdly, as noted by Yang and Chen (2023), many of these methods struggle to control the false discovery rate (FDR). Consequently, there is a pressing need for a method that is distribution-free, powerful, and robust (i.e., with well-controlled FDR), while also accommodating the correlation structure present in longitudinal data.

We introduce ZINQ-L, a zero-inflated quantile regression approach for DA analysis of longitudinal microbiome data. ZINQ-L extends the ZINQ framework (Ling et al., 2021) by incorporating adjustments for within-subject correlations in longitudinal studies. In the first component, a logistic mixed-effects regression models the zero inflation inherent in microbiome data, and the second component involves quantile mixed-effects regression models the nonzero data. Investigating multiple quantiles of the nonzero part, we utilize a quantile rank-score test adjusted for zero inflation to derive p-values, which are then integrated with the p-value from the logistic mixed-effects model to form the final inference. Unlike traditional methods, our approach does not rely on specific distributional assumptions and is compatible with various normalization procedures. Extensive simulations demonstrate that ZINQ-L achieves well-controlled type I error, robust FDR control, and superior or comparable power relative to existing methods. Furthermore, ZINQ-L is capable of detecting heterogeneous associations arising from complex mechanisms beyond mere mean shifts. We applied ZINQ-L to a longitudinal kidney transplant cohort, successfully identifying promising novel taxa associated with antibiotic treatments, beyond conventional analysis which we have previously performed (Dong et al., 2024).

## 2 Methods

Consider a longitudinal microbiome study comprising m subjects, with each subject i providing $n_{i}$ repeated measurements. Consequently, the total number of observations across all subjects is $N=\sum_{i=1}^{m}n_{i}$ . The resulting microbiome data is organized into a count table with dimensions N×K , where K represents the number of microbial taxa (amplicon sequence variants, operational taxonomic units, species, genera, or other taxonomic level). For each sample from subject i and visit j , we let $Y_{\mathrm{ijk}}$ denote the normalized abundance (relative abundance, rarefied count, etc.) of taxon k , and let $S_{ij}={\left(X_{ij},Z_{ij}^{⊤}\right)}^{⊤}$ denote the covariates, where $X_{ij}$ is the scalar phenotype of interest (continuous or dichotomous exposure or outcome) and $Z_{ij}={\left(1,Z_{\mathrm{ij1}},\ldots ,Z_{ij\left(q-1\right)}\right)}^{⊤}$ is a q×1 vector of adjusting covariates including the intercept. Because ZINQ-L analyzes individual taxa independently, we will omit the subscript k in the rest of the paper for simplicity.

### 2.1 Two-part quantile regression model for longitudinal data

To model the zero-inflated taxon abundance, we decompose the conditional distribution as

$$F\left(Y_{ij}|S_{ij}\right)=P\left(Y_{ij}=0|S_{ij}\right)+F\left(Y_{ij}|S_{ij},Y_{ij}>0\right)P\left(Y_{ij}>0|S_{ij}\right),$$

and model the two components, $P\left(Y_{ij}>0|S_{ij}\right)$ , the probability of presence in subject i and visit j , and $F\left(Y_{ij}|S_{ij},Y_{ij}>0\right)$ , the conditional distribution of abundance given the taxon is present in subject i and visit j , separately.

In the first part, we assume the probability of presence to follow a logistic mixed-effects model that accounts for the within-subject correlations,

$$\text{logit}\left\{P\left(Y_{ij}>0|S_{ij}\right)\right\}=\gamma X_{ij}+Z_{ij}^{⊤}\xi +h_{i}^{L}$$

where γ,ξ are fixed effects associated with the interested phenotype and covariates, and $h_{i}^{L}∼N{\left(0,\sigma_{h}^{L}\right)}^{2}$ is the subject-specific random intercept.

In the second part, as an alternative to mean-based methods that rely on parametric assumptions, a non-parametric quantile-based model is assumed for the non-zero abundance $Y_{ij}|Y_{ij}>0$ . To account for within-subject correlations, we add the random effect such that

$$Y_{ij}|Y_{ij}>0=\beta X_{ij}+Z_{ij}^{⊤}\alpha +h_{i}+\epsilon_{ij},$$ (1)

where $h_{i}$ is the subject-specific random intercept without distributional assumptions, and the error term $\epsilon_{ij}$ has no distributional assumptions as well. We adopt the marginal longitudinal quantile regression model (Wang and He, 2007), which defines $u_{ij}=h_{i}+\epsilon_{ij}$ as the composite error and assumes the τ th conditional quantile of $u_{ij}$ is zero, i.e., $Q_{u_{ij}}\left(\tau |S_{ij},Y_{ij}>0\right)=0$ , to avoid identifiability issues. Since no distributional assumptions are made for $u_{ij}$ and Equation 1 is quantile-specific, i.e., the quantile coefficients and error can be represented as β(τ) , α(τ) , and $u_{ij}\left(\tau \right)$ and change with the quantile level τ , we can rewrite the quantile part as

$$Q_{Y_{ij}}\left(\tau |S_{ij},Y_{ij}>0\right)=\beta \left(\tau \right)X_{ij}+Z_{ij}^{⊤}\alpha \left(\tau \right),$$

where β(τ),α(τ) are fixed effects associated with the interested phenotype and covariates at the τ ’s conditional quantile of the non-zero abundance, e.g., the conditional median, $Q_{Y_{ij}}\left(0.50|S_{ij},Y_{ij}>0\right)$ , or the third conditional quartile, $Q_{Y_{ij}}\left(0.75|S_{ij},Y_{ij}>0\right)$ . If $Y_{ij}$ is a count variable such as the rarefied count, to break ties and achieve valid inference, we add a perturbation $W_{ij}=Y_{ij}+U,U∼U\left(0,1\right)$ , and model the conditional quantiles of $W_{ij}$ instead. This is the standard technique to apply quantile regression for counts (Machado and Silva, 2005). β(τ) and α(τ) can then be estimated by

$$\min_{\beta ,\alpha }\overset{m}{\underset{i=1}{\sum }}\overset{n_{i}}{\underset{j=1}{\sum }}\rho_{\tau }\left(Y_{ij}-\beta X_{ij}-Z_{ij}^{⊤}\alpha \right)\cdot I\left(Y_{ij}>0\right),$$

where $\rho_{\tau }\left(u\right)=u\left\{\tau -I\left(u<0\right)\right\}$ is the quantile loss function (Koenker and Bassett Jr, 1978). Though the model specification with the composite error seems to be the same as the cross-sectional quantile regression model, the testing procedure will incorporate within-subject correlations (Section 2.2).

In DA analysis, our goal is to identify individual taxa whose abundance varies according to the variable of interest over time, which, based on the two-part longitudinal model, can be decomposed into whether the taxon’s presence-absence status is associated with X over time (captured by γ ) and whether the distribution of abundance is associated with X over time given the presence of the taxon (captured by β(τ), ∀τ∈(0,1) ). Mathematically, we can formulate the DA analysis into the following hypothesis testing.

$$H_{0}:\gamma =0\;\text{and}\;\beta \left(\tau \right)=0,\;\forall \tau \in \left(0,1\right),$$ (2)

$$H_{1}:\gamma \neq 0\;\text{or}\;\exists \tau^{*}\in \left(0,1\right),\;\text{s.t.}\;\beta \left(\tau^{*}\right)\neq 0.$$

To test γ=0 , the Wald test or likelihood ratio test (LRT) is readily available for the logistic mixed-effects regression. Here, we choose LRT to achieve a better finite-sample power (Paek, 2009; Hauck and Donner, 1977). However, no existing methods can be directly applied to test β(τ)=0 under the zero-inflated longitudinal quantile regression model. Therefore, we propose a quantile rank-score test that accounts for both the longitudinal structure and zero inflation (Section 2.2). To test β(τ)=0,∀τ∈(0,1) , we conduct the proposed test at multiple quantile levels, $0<\tau_{1}<\tau_{2}<\cdots <\tau_{L}<1$ , which cover the entire distribution of the non-zero part and a usual pick could be τ=0.10,0.25,0.50,0.75,0.9 . Finally, we use an omnibus test (Section 2.3) to combine the marginal tests, including both the longitudinal logistic test and the series of longitudinal quantile tests, and obtain the final p-value that indicates whether the taxon’s abundance distribution is differential according to the interested phenotype over time.

### 2.2 Zero-inflated quantile rank-score test for longitudinal microbiome data

Existingtools for longitudinal quantile regression are not suitable for microbiome studies. Some approaches ignore the zero inflation, which can lead to an underestimation of the uncertainty associated with observing non-zero outcomes, resulting in biased results (Wang and He, 2007). Others (Wang and Fygenson, 2009) address this issue by analyzing the underlying unconstrained outcomes within a censored longitudinal quantile regression framework but do not model the presence-absence status. These approaches are inapplicable to microbiome data because both the presence-absence status and the distribution of non-zero values are of analytical interest. To bridge this methodological gap, we propose an advanced rank-score test for β(τ)=0 within a two-part quantile regression model for longitudinal data. This novel approach adeptly adjusts for zero inflation, enhancing the accuracy and reliability of inferences in scenarios where zero-inflated data are prevalent.

Since the quantile regression part is restricted to the non-zero abundances, we let ${\tilde{X}}_{ij}=X_{ij}\cdot I\left(Y_{ij}>0\right)$ and ${\tilde{Z}}_{ij}=Z_{ij}\cdot I\left(Y_{ij}>0\right)$ denote the nominal variables of the interested phenotype and the adjusting covariates. It follows that ${\tilde{X}}_{N\times 1}={\left({\tilde{X}}_{11},{\tilde{X}}_{12},\ldots ,{\tilde{X}}_{1n_{1}},\ldots ,{\tilde{X}}_{m1},{\tilde{X}}_{m2},\ldots ,{\tilde{X}}_{mn_{m}}\right)}^{⊤}$ and ${\tilde{Z}}_{N\times q}={\left({\tilde{Z}}_{11},{\tilde{Z}}_{12},\ldots ,{\tilde{Z}}_{1n_{1}},\ldots ,{\tilde{Z}}_{m1},{\tilde{Z}}_{m2},\ldots ,{\tilde{Z}}_{mn_{m}}\right)}^{⊤}$ are the design vector and matrix associated with ${\tilde{X}}_{ij}$ ’s and ${\tilde{Z}}_{ij}$ ’s. We denote ${\tilde{X}}^{*}=\left(I-P_{Z}\right)\tilde{X}$ , where $P_{Z}=\tilde{Z}{\left({\tilde{Z}}^{⊤}\tilde{Z}\right)}^{-1}{\tilde{Z}}^{⊤}$ and I is the N×N identity matrix. This orthogonal transformation ensures the asymptotic independence between ${\tilde{X}}^{*}$ and $\tilde{Z}$ .

We construct a quantile rank score for β(τ)=0 by

$$S_{N}\left(\tau \right)=N^{-\frac{1}{2}}\overset{m}{\underset{i=1}{\sum }}\overset{n_{i}}{\underset{j=1}{\sum }}{\tilde{X}}_{ij}^{*}\psi_{\tau }\left\{{\hat{u}}_{ij}\left(\tau \right)\right\}I\left(Y_{ij}>0\right),$$

where $\psi_{\tau }\left(u\right)=\tau -I\left(u<0\right)$ is the score function, which is the piecewise first derivative of the quantile loss function $\rho_{\tau }\left(u\right)$ , ${\hat{u}}_{ij}\left(\tau \right)=Y_{ij}-{\tilde{Z}}_{ij}^{⊤}\hat{\alpha }$ is the residual of (4) under the null with β=0 , and ${\tilde{X}}_{ij}^{*}$ is the element of ${\tilde{X}}^{*}$ corresponding to the i th subject and the j th measurement. By design, $S_{N}\left(\tau \right)$ measures the independent contribution of X onto the τ th quantile of Y|Y>0 , which is close to 0 when β(τ)=0 and its deviation from 0 indicates associations. Letting

$$Q_{N}\left(\tau \right)=N^{-1}\tau \left(1-\tau \right)\overset{m}{\underset{i=1}{\sum }}\overset{n_{i}}{\underset{j=1}{\sum }}{{\tilde{X}}_{ij}^{*}}^{2}+N^{-1}\overset{m}{\underset{i=1}{\sum }}\underset{j_{1}\neq j_{2}}{\sum }{\tilde{X}}_{ij_{1}}^{*}{\tilde{X}}_{ij_{2}}^{*}{\hat{\delta }}_{ij_{1}j_{2}},$$ (3)

where ${\hat{\delta }}_{ij_{1}j_{2}}=\tau^{2}I\left(Y_{ij_{1}}>0,Y_{ij_{2}}>0\right)-2\tau I\left\{{\hat{u}}_{ij_{1}}\left(\tau \right)<0,Y_{ij_{1}}>0,Y_{ij_{2}}>0\right\}+I\left\{{\hat{u}}_{ij_{1}}\left(\tau \right)<0,{\hat{u}}_{ij_{2}}\left(\tau \right)<0,Y_{ij_{1}}>0,Y_{ij_{2}}>0\right\}$ , we can have the τ th quantile rank-score test statistics and its asymptotic distribution under mild conditions (Appendix) such that as m→∞ therefore N→∞ ,

$$T_{N}\left(\tau \right)=S_{N}^{2}\left(\tau \right)/Q_{N}\left(\tau \right)\overset{d}{\to }\chi_{1}^{2}.$$

We note that the second term of Equation 3 accounts for the correlation within a subject, which is estimated block-wisely within each subject and then averaged across the subjects. Different from Ling et al. (2021), Wang and He (2007), Wang and Fygenson (2009), the proposed test simultaneously accommodates the within-subject correlations of longitudinal data and the two-part framework for zero-inflated microbiome data.

Similarly, we can obtain the asymptotic joint distribution of quantile rank scores at multiple τ ’s, $W_{N}={\left(S_{N}\left(\tau_{1}\right),S_{N}\left(\tau_{2}\right),\ldots ,S_{N}\left(\tau_{L}\right)\right)}^{⊤}$ , which is useful for combining the marginal tests. Given $\beta \left(\tau_{1}\right)=\beta \left(\tau_{2}\right)=\cdots =\beta \left(\tau_{L}\right)=0$ and m→∞ therefore N→∞ , $W_{N}\overset{d}{\to }N\left(0,V\right)$ , where V can be estimated by $V_{N}=\left(v_{N}^{\left(ab\right)}\right)$ such that $v_{N}^{\left(ab\right)}=N^{-1}\left(\min\left\{\tau_{a},\tau_{b}\right\}-\tau_{a}\tau_{b}\right)\sum_{i=1}^{m}\sum_{j=1}^{n_{i}}{\tilde{X}}_{ij_{2}}^{*}+N^{-1}\sum_{i=1}^{m}\sum_{j_{1}\neq j_{2}}{\tilde{X}}_{ij_{1}}^{*}{\tilde{X}}_{ij_{2}}^{*}{\hat{\delta }}_{ij_{1}j_{2}}\left(\tau_{a},\tau_{b}\right)$ and ${\hat{\delta }}_{ij_{1}j_{2}}\left(\tau_{a},\tau_{b}\right)=\tau_{a}\tau_{b}I\left(Y_{ij_{1}}>0,Y_{ij_{2}}>0\right)-\tau_{b}I\left(u_{ij_{1}}\left(\tau_{a}\right)<0,Y_{ij_{1}}>0,Y_{ij_{2}}>0\right)-\tau_{a}I\left(u_{ij_{2}}\left(\tau_{b}\right)<0,Y_{ij_{1}}>0,Y_{ij_{2}}>0\right)$ $+I\left(u_{ij_{1}}\left(\tau_{a}\right)<0,u_{ij_{2}}\left(\tau_{b}\right)<0,Y_{ij_{1}}>0,Y_{ij_{2}}>0\right)$ .

### 2.3 Omnibus test for marginal tests combination

Finally, to obtain a single p-value for testing the null hypothesis Equation 2, which indicates whether there is a differential distribution of the taxon abundance over time, we conduct an omnibus test that combines the marginal longitudinal logistic and quantile tests.

As discussed above, we conduct LRT, $T^{L}$ , for $\beta^{L}=0$ under the logistic mixed-effects regression model (2) and obtain a p-value $p^{L}$ , and then conduct the proposed zero-inflated quantile rank-score test for longitudinal data at multiple quantile levels, $T_{N}\left(\tau_{i}\right),\;0<\tau_{1}<\tau_{2}<\cdots <\tau_{l}<1$ , for β(τ)=0,∀τ∈(0,1) under the quantile mixed-effects regression model (4) and obtain p-values $p_{\tau_{1}},p_{\tau_{2}},\ldots ,p_{\tau_{L}}$ . To combine them together, we use either the MinP procedure or the truncated Cauchy combination test.

The MinP test (Lee et al., 2012; He et al., 2017) picks the smallest p-value from $p^{L},p_{\tau_{1}},p_{\tau_{2}},\ldots ,p_{\tau_{L}}$ as the test statistic, and rejects the null hypothesis if it is unlikely to observe an even smaller minimum p-value under the null. Specifically, the omnibus p-value is computed by.

$$\begin{array}{ll} p^{\mathrm{MinP}} & =P\left(\min\left(p^{L},p_{\tau_{1}},p_{\tau_{2}},\ldots ,p_{\tau_{L}}\right)\leq p^{\mathrm{obs}}|H_{0}\right)\\ & =1-P\left(p^{L}>p^{\mathrm{obs}},p_{\tau_{l}}>p^{\mathrm{obs}}\;\;\forall l=1,\ldots ,L|H_{0}\right)\\ & =1-P\left(p^{L}>p^{\mathrm{obs}}|H_{0}\right)\times P\left(p_{\tau_{l}}>p^{\mathrm{obs}}\;\;\forall l=1,\ldots ,L|H_{0}\right) \end{array}$$ (4)

$$=1-\left(1-p^{\mathrm{obs}}\right)\times P\left(T_{N}\left(\tau_{l}\right)\leq Q_{\chi_{1}^{2}}\left(1-p^{\mathrm{obs}}\right)\;\forall l=1,\ldots ,L\right),$$ (5)

where Equation 4 is due to the conditional independence between $T^{L}$ and $T_{N}\left(\tau_{l}\right)$ ’s, (Equation 5) is based on the fact that $p^{L}∼U\left(0,1\right)$ and the asymptotic distribution of $T_{N}\left(\tau_{l}\right)$ ’s under the null, and $P\left(T_{N}\left(\tau_{i}\right)\leq Q_{\chi_{1}^{2}}\left(1-p^{\mathrm{obs}}\right)\;\forall i=1,\ldots ,L\right)$ can be estimated by resampling the joint limiting distribution of $W_{N}$ under the null.

The truncated Cauchy combination test (Fang et al., 2023) computes a weighted sum of the tangent-transformed p-values as the test statistic while taking special care of extreme p-values. Specifically,

$$T_{\text{ZINQ}-\text{L tCauchy}}={\hat{r}}_{n}g\left(p^{L}\right)+L^{-1}\left(1-{\hat{r}}_{n}\right)\overset{L}{\underset{l=1}{\sum }}g\left(p_{\tau_{l}}\right),$$

where $g\left(p\right)={\left(p\pi \right)}^{-1}\cdot I\left(p<10^{-15}\right)+\tan\left\{\left(0.5-p\right)\pi \right\}\cdot I\left(10^{-15}\leq p\leq \delta \right)+\tan\left\{\left(0.5-\delta \right)\pi \right\}\cdot I\left(p>\delta \right)$ and δ→1 , and ${\hat{r}}_{n}$ is the zero rate of the investigated taxon. We note that for extremely small p-values, their transformation is approximated by the first term of the Taylor expanded tangent transformation, while for the extremely large p-values, they are truncated first by a predefined threshold δ before the tangent transformation, where δ is usually set to be 0.99. The omnibus p-value can be computed given that $T_{\text{ZINQ}-\text{L tCauchy}}$ converges to the standard Cauchy distribution under the null. This truncated Cauchy combination approach (Fang et al., 2023) has been shown to be more powerful than the classic Cauchy combination approach (Liu and Xie, 2020) when some of the individual p-values are very close to one.

In general, ZINQ-L MinP is more rigorous than ZINQ-L tCauchy as it leverages the correlation structure between the marginal tests. By design, ZINQ-L tCauchy performs well primarily at the tail. However, since ZINQ-L tCauchy avoids resampling from the joint limiting distribution, it is more computationally efficient, making it appealing for large-scale data analysis.

## 3 Overview of KTx data

The Kidney Transplant study (KTx) (Magruder et al., 2019) aims to investigate the association between gut microbiota and post-transplant complications among the immunosuppressed kidney transplant recipients. It comprises 510 fecal samples collected from 168 kidney transplant recipients in the first 3 months after transplantation between August 2015 and November 2016. Each recipient provided between 1 and 6 fecal samples, with an average of 3 samples per individual. These samples underwent gut microbiome profiling via 16S rRNA gene sequencing of the V4-V5 hypervariable region. Patient-level characteristics were also collected, covering demographic information such as gender and age, transplant-related information such as prior transplantation history, and granular therapy data such as antibiotic usage.

We filtered out samples with less than 10,000 total counts of microbes and rarefied the remaining samples to 10,000 library size, resulting in 429 samples from 160 patients. Our primary variable of interest is antibiotic treatment within the first 120 days after transplantation, in addition to preoperative antibiotic prophylaxis and *Pneumocystis jirovecii* (PJP) prophylaxis. We treated antibiotic (denoted by Abx) administrations as a time-ever event. For example, if a kidney transplant recipient had repeated measurements of gut microbiota at post-transplant day 30, 45, and 60 and antibiotic treatment at post-transplant day 40, the antibiotic exposures at the 3 measurements were then defined as No Abx, Abx, and Abx. In total, there were 117 samples exposed to antibiotics and 312 samples without prior antibiotic administrations, with 61 patients having at least one measurement exposed to the treatment. The average age in Abx samples is 54 and that in No Abx samples is 53. There is a higher proportion of female in the Abx group (60%) than in the No Abx group (39%), with a Fisher’s exact test p-value < 0.001, suggesting that gender can be a potential confounder.

We aggregated the microbiome data to the genus level and removed the rare genera that were present in less than 90% of the samples. The final processed data contained 119 taxa.

## 4 Simulation experiments

We conducted extensive simulations to evaluate the performance of ZINQ-L compared to commonly used competing methods. The simulations were based on the filtered and rarefied KTx data (Section 3). The starting dataset comprised the most recent visits of 143 patients who had multiple visits. It is worth noting that we restricted the starting data to contain only a single sample per patient, thus the 143 patients were independent. Of these 143 patients, those with any prior antibiotic treatment before their last visit were labeled as Abx (n = 54), while the remainder were labeled as No Abx (n = 89). We excluded rare taxa that were present in less than 10% of the patients, resulting in a final dataset containing 118 taxa. In both Simulation 1 and 2, we generated data with different numbers of subjects, m=200,500 , by sampling the 143 patients with replacement. For each subject, we then expanded the single observation to different numbers of visits, $n_{i}=3,5,7,9$ .

### 4.1 Simulation 1 - unadjusted analysis on individual taxon

**FIGURE 1:**
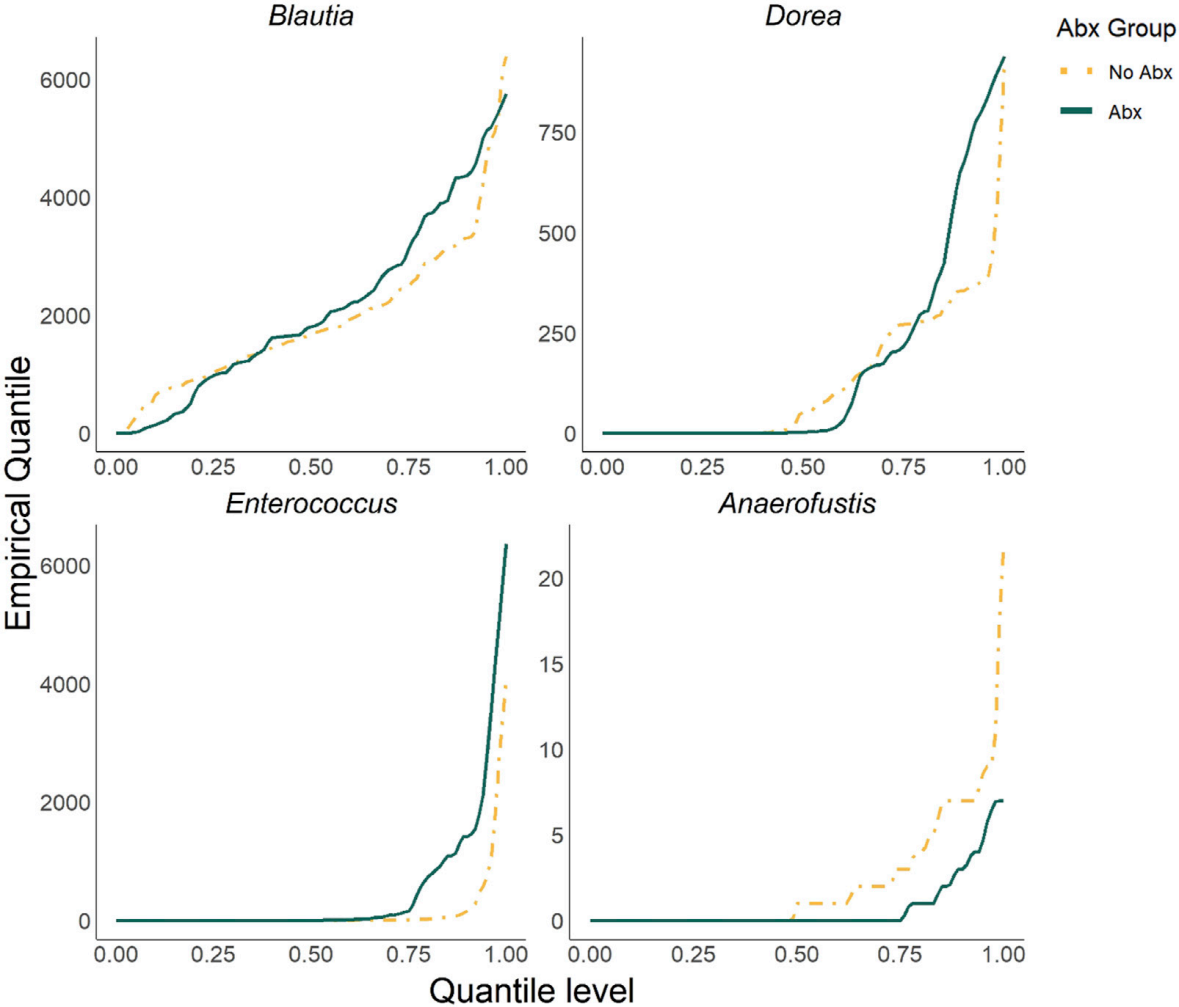
The plot of empirical quantiles (stratified by Abx and No Abx patients) of the four representative taxa selected from the KTx-based starting data for Simulation 1.

We first aimed to evaluate the performance of ZINQ-L at the individual taxa level by investigating the association between four representative taxa and Abx treatment in a longitudinal setting, without adjusting for other covariates. The four selected taxa were *Blautia*, *Dorea*, *Enterococcus*, and *Anaerofustis*, all of which were differentially abundant between the Abx and No Abx groups based on the starting data. Specifically, the mean differences for *Blautia* and *Dorea* were minimal (two-sample t-test p-values = 0.4637 and 0.3643) while their distributional differences were apparent when examined through their empirical quantiles (Figure 1). In contrast, *Enterococcus* and *Anaerofustis* primarily exhibited (marginal) mean differences (two-sample t-test p-values = 0.0573 and 0.0019). The zero inflation rates for *Blautia*, *Dorea*, *Enterococcus*, and *Anaerofustis* were approximately 3%, 42%, 48%, and 59%, respectively, representing a range from common to relatively rare taxa. It is worth noting that three of the four taxa, *Blautia*, *Dorea*, and *Enterococcus*, are important bacteria in antibiotic treatment literature (Jenq et al., 2015; Shi et al., 2018; Ubeda et al., 2010), highlighting the real-world relevance of this simulation study.

We first sampled m (200 or 500) subjects based on each of the four taxa observations from the KTx-based starting data. For the type I error assessment, m subjects were randomly sampled with replacement from a mixture of Abx and No Abx patients, and then m/2 of them were randomly assigned to the Abx group. This procedure ensured that the abundance of the representative taxa was not differentiated between the Abx and No Abx subjects. For the power assessment, m/2 subjects were randomly sampled with replacement from the Abx group (n = 54), while the remaining m/2 subjects were randomly sampled with replacement from the No Abx group (n = 89). This approach preserved the associations between the abundance of the taxa and antibiotic treatment in the simulated data.

Next, longitudinal effects were introduced to the m independent subjects obtained for either the type I error or power assessment. Let $A_{i}$ denote the microbial count of subject i . The $n_{i}$ repeated measures were then generated by expanding and perturbing $A_{i}$ . Specifically, the taxonomic count $A_{ij}$ for subject i at visit j was generated using the formula $A_{ij}=\exp\left\{\log\left(A_{i}+1\right)+\epsilon_{ij}\right\}-1$ , where $j=1,\ldots ,n_{i}$ and i=1,…,m , with the resulting value rounded to the nearest integer. If the resulting $A_{ij}$ was smaller than zero, we assigned it as zero. The random perturbation $\epsilon_{ij}$ followed a standard normal distribution. The log-exponentiation transformation ensured non-negative microbial counts, while a pseudo-count of 1 (subtracted after exponentiation) was added to avoid zeros in the logarithm. This procedure was repeated $n_{i}$ times to represent $n_{i}$ visits for each subject, ensuring that the repeated measurements for the same subject were correlated, with variations over time incorporated. The Abx status for each subject remained consistent across multiple visits. No other covariates were considered in this simulation.

In addition to ZINQ-L tCauchy and ZINQ-L MinP, we applied three competing methods to the simulated data: LMM, zero-inflated Gaussian mixed model (ZIGMM), and ZINBMM, with the latter two from the NBZIMM package (Zhang and Yi, 2020). MaAsLin2 (Mallick et al., 2021) and LinDA (Zhou et al., 2022) were excluded from this simulation as they are only applicable to OTU tables, not individual taxa. Furthermore, since both are linear model-based approaches, LMM served as a representative for their kind.

The simulation was conducted independently for each of the four representative taxa. In each simulation run, differential abundance over time was identified if the corresponding p-value was less than 0.05. We assessed type I error control on the null data by calculating the percentage of differentially abundant cases over 10,000 runs and evaluated power on the alternative data by the proportion of positive calls among 2,000 replicates.

### 4.2 Simulation 2 - adjusted analysis on OTU table with partial null and alternative (FDR control)

The second simulation aims to mimic a real OTU count table, where all taxa are analyzed against the phenotype of interest with covariates adjusted, while only a subset of the taxa are truly associated. By applying ZINQ-L to these simulated OTU tables, we can evaluate whether ZINQ-L effectively controls the false discovery rate (FDR) while maintaining a reasonable true positive rate (TPR).

We simulated the read counts for each taxon in the community using a zero-inflated quantile regression model and then combined them to form a community. For each of the 118 taxa in the KTx-based starting data, we fitted the zero-inflated quantile regression model with two covariates: Abx (the key variable of interest) and Age (the adjusting covariate). The logistic regression component obtained was: $\text{logit}\left\{P\left(D_{i}=1|S_{i}\right)\right\}=\hat{\gamma }{\text{Abx}}_{i}+\hat{\xi_{0}}+\hat{\xi_{1}}{\text{Age}}_{i}$ , where $D_{i}=I\left(Y_{i}>0\right)$ represented the presence-absence status, Age was normalized before fitting, and $\hat{\gamma },\hat{\xi_{0}},\hat{\xi_{1}}$ were the fitted coefficients. The quantile regression component obtained was: $Q_{Y_{i}}\left(\tau |S_{i},Y_{i}>0\right)=\hat{\beta }\left(\tau \right){\text{Abx}}_{i}+\hat{\alpha_{0}}\left(\tau \right)+\hat{\alpha_{1}}\left(\tau \right){\text{Age}}_{i}$ , where τ=0.01,…,0.99 , and the fitted coefficient functions, $\hat{\beta }\left(\tau \right),\hat{\alpha_{0}}\left(\tau \right),\hat{\alpha_{1}}\left(\tau \right)$ , were interpolated to ensure that the entire abundance distribution, given the taxon’s presence, could be generated in the simulation step.

Next, before data generation, we categorized the 118 taxa into rare and common groups based on their prevalence in the starting data. The categorization resulted in 60 rare taxa (average zero inflation rate of 91%) and 58 common taxa (average zero inflation rate of 40%). We conducted three simulation scenarios: (1) only the common taxa were differentially abundant, (2) only the rare taxa were differentially abundant, and (3) a randomly selected mixture of 30 rare taxa and 29 common taxa was differentially abundant.

We first simulated the covariates for m (200 or 500) subjects, with each subject having $n_{i}$ repeated visits $\left(n_{i}=3,5,7,9\right)$ . We initiated by simulating covariates (Abx status and Age) for the first visits. From the starting data based on the KTx study, we randomly sampled Abx status and initial Age for m subjects with replacement. For each subsequent visit j , the Abx status remained constant while the Age increased by 0.1 per follow-up. Note that Age was normalized in the original study to have mean 0 and variance 1.

Next, we simulated the microbiome OTU tables. For each taxon, we generated the binary variable $D_{ij}$ for visit j of subject i , indicating the taxon’s presence-absence. $D_{ij}$ is derived from a Bernoulli distribution with probability $p_{ij}$ , where $\text{logit}\left(p_{ij}\right)=\gamma {\text{Abx}}_{ij}+{\hat{\xi }}_{0}+{\hat{\xi }}_{1}{\text{Age}}_{ij}+h_{i}^{L}$ and $h_{i}^{L}∼N\left(0,1\right)$ . Here, we set γ=0 if the taxon’s presence or absence was not associated with Abx, and $\gamma =\hat{\gamma }$ , the estimate from the starting data without repeated measures, for each associated taxon. Following this setting, if $D_{ij}=0$ , we assigned $Y_{ij}=0$ . If $D_{ij}=1$ , we simulated $Y_{ij}$ using the inverse cumulative distribution function method. Specifically, we randomly drew $U_{ij}∼U\left(0,1\right)$ , and then generated $Y_{ij}=\beta \left(U_{ij}\right){\text{Abx}}_{ij}+\hat{\alpha_{0}}\left(U_{ij}\right)+\hat{\alpha_{1}}\left(U_{ij}\right){\text{Age}}_{ij}+h_{i}$ with $h_{i}∼N\left(0,1\right)$ , and rounded $Y_{ij}$ to the nearest integer. Again, if this taxon was in the null set, we set β(τ)=0,τ∈(0,1) , and when the taxon was in the alternative set, we used its corresponding fitted value $\hat{\beta }\left(\tau \right),\tau \in \left(0,1\right)$ based on the starting data. The simulated taxa were then concatenated to form an OTU table for analysis.

In addition to the proposed ZINQ-L, we applied the competing methods LMM, ZIGMM, ZINBMM, LinDA, and MaAsLin2 to the simulated OTU table. The resulting p-values were adjusted using the Benjamini–Hochberg (BH) procedure (Benjamini and Hochberg, 1995). A taxon was considered differentially abundant over time if its adjusted p-value was less than 0.05. Accordingly, FDR was calculated as the proportion of rare taxa detected among all detected taxa, and TPR was calculated as the percentage of common taxa detected. The simulation was repeated 1,000 times. To evaluate the performance of the different methods, we computed the average FDR and TPR over the 1,000 runs.

## 5 Simulation results

### 5.1 Result of simulation 1

**FIGURE 2:**
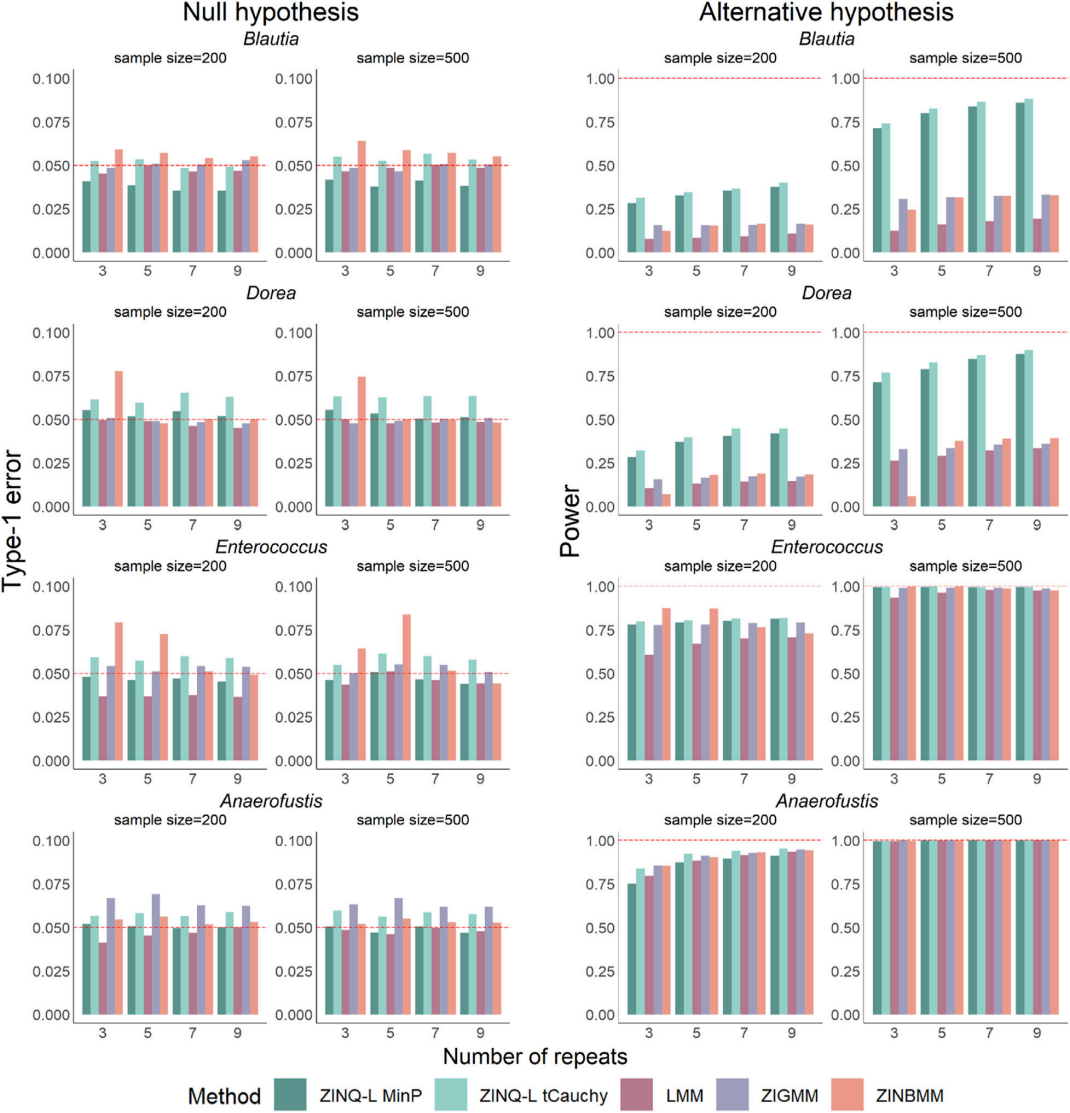
Type I error and power by unadjusted analysis on simulated individual taxa. The left panels show the type I error under the null cases, while the right panels show the power under alternative cases. Each row corresponds to each of the four representative taxa. Various samples sizes and number of visits were investigated.

Regarding type I error control (Figure 2, left panels), LMM effectively controlled type I error in all settings, followed by ZINQ-L MinP, which adhered to the nominal level of 0.05 in most cases. ZINQ-L tCauchy showed slight inflation across all settings. However, ZINQ-L tCauchy demonstrated preferable computational efficiency compared to ZINQ-L MinP (Table 1) and valid FDR control (see Simulation 2), making it suitable for large-scale data analysis.

**TABLE 1:** Computation time (min) to analyze simulated OTU tables and KTx data 10 times.

Dataset	Simulated OTU table								KTx
m	200				500				—
$n_{i}$	3	5	7	9	3	5	7	9	—
LMM	0.83	0.89	0.96	1.02	1.2	1.34	1.42	1.43	0.64
LinDA	0.85	0.92	0.99	1.04	1.23	1.38	1.43	1.5	0.76
MaAsLin2	1.01	1.11	1.2	1.29	1.51	1.74	1.85	1.93	0.89
ZINBMM	8.32	8.62	10.62	12.59	17.71	18.73	21.0	25.69	8.9
ZIGMM	4.97	5.63	6.03	6.5	10.45	11.21	12.31	13.84	4.79
ZINQ-L MinP	13.18	16.46	19.17	21.78	50.01	54.24	65.7	74.13	11.37
ZINQ-L tCauchy	10.71	13.69	16.12	18.61	47.06	51.44	59.52	66.58	9.36

ZINBMM showed pronounced type I error inflation for *Blautia*, *Dorea*, and *Enterococcus*, while ZIGMM exhibited type I error inflation for *Anaerofustis*, particularly when the number of repeated measures was small. The consistent robustness of ZINQ-L can be attributed to its non-parametric nature, making it resilient to the complex distributions of taxa abundances.

For the power assessment (Figure 2, right panels), all methods exhibited increased power as the sample size (m) and the number of visits $\left(n_{i}\right)$ increased, demonstrating the consistency of all approaches. Notably, both ZINQ-L tCauchy and ZINQ-L MinP outperformed the competitors in detecting differences for *Blautia* and *Dorea*, while showing comparable performance for the remaining two taxa where mean differences were present. This indicates that ZINQ-L excels in identifying distributional differences, particularly when crossing effects occur. The improved power of ZINQ-L arises from its comprehensive examination of differences at multiple locations of the abundance distribution. As Figure 1 illustrates, *Blautia* and *Dorea* are depleted with Abx at lower quantiles but enriched at upper quantiles. However, the mean difference, which is the integrated effect across the entire distribution, is cancelled out. Unlike the mean-based competitors, the quantile-based ZINQ-L can identify and aggregate signals at different quantiles rather than relying solely on mean shifts.

Overall, ZINQ-L is a robust method that effectively controls type I error and demonstrates comparable or improved power in detecting longitudinal associations when mean or quantile differences are present for the key variable of interest.

### 5.2 Result of simulation 2

**FIGURE 3:**
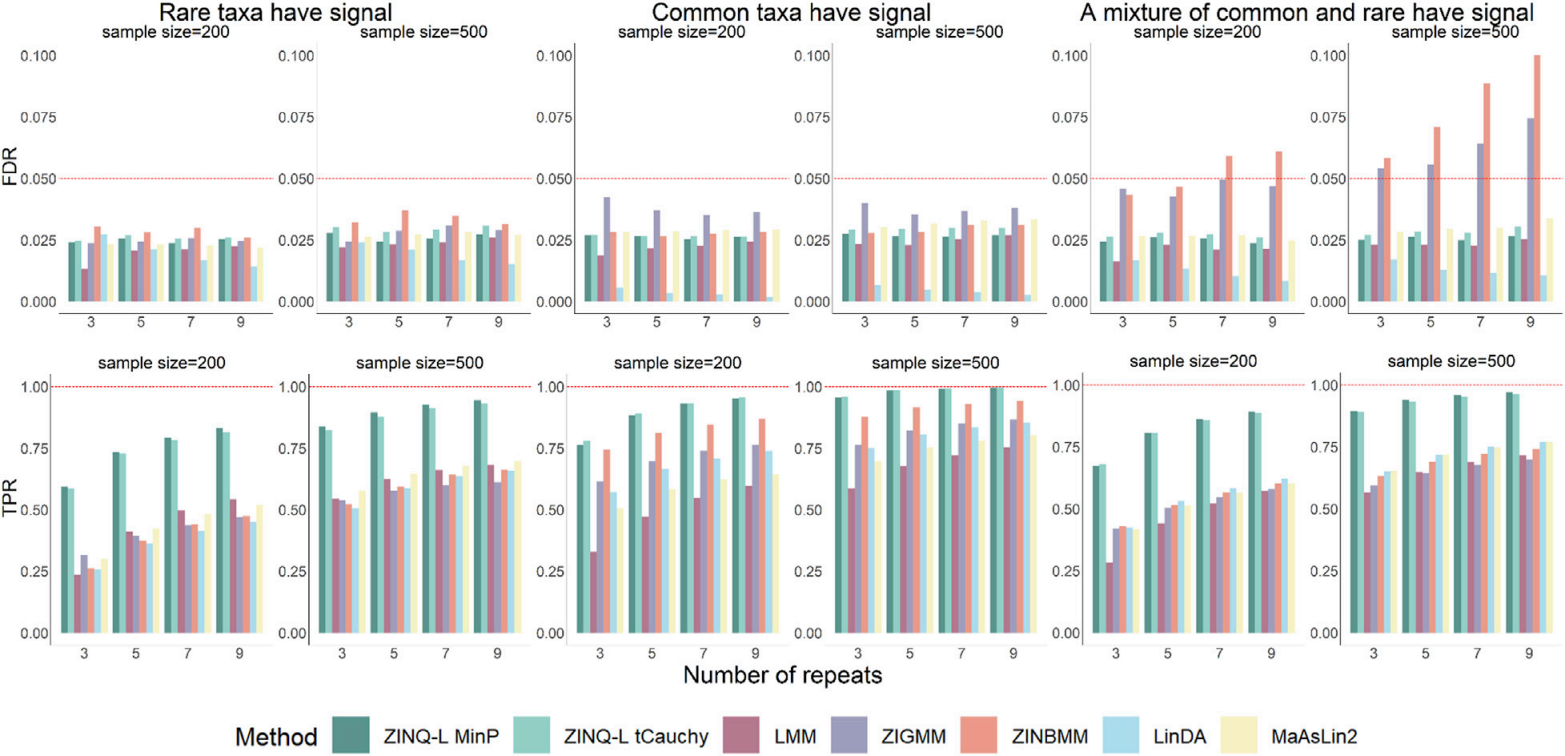
FDR and TPR for by adjusted analysis on simulated OTU tables. The top panels show FDR, while the bottom panels show TPR. The left, middle, and right panel represent the scenarios where the rare taxa, the common taxa, or half of the common taxa as well as half of the rare were simulated to be differentially abundant, respectively.

The upper panels of Figure 3 present the FDR results across all scenarios in Simulation 2. In situations where the differentially abundant taxa were either all common or rare, all tested methods successfully controlled the FDR below the 0.05 threshold. However, in scenarios where a mixture of common and rare taxa were differentially abundant, ZIGMM and ZINBMM reported inflated FDRs, especially as the sample size or the number of repeated measurements increased. Conversely, all other methods appropriately controlled the FDR.

Further analysis was conducted to identify the cause of the FDR inflation in mixed-taxa scenarios. FDR was calculated separately for the common and rare taxa within this simulation scenario. As illustrated in Supplementary Figure S1, this FDR inflation is primarily driven by the FDR inflation among the common taxa. This suggests that the incorrect distributional assumptions of ZIGMM and ZINBMM make them sensitive to the varying signal profiles within the microbiome community.

Notably, ZINQ-L tCauchy did not inflate the FDR when analyzing the entire OTU table with different scenarios of differential abundant taxa. This suggests that ZINQ-L tCauchy can enhance its robustness by averaging over heterogeneous signals. Additionally, this finding alleviates concerns about its slight type I error inflation observed in Simulation 1, as in real-world analysis, researchers typically work with OTU tables and use FDR control to evaluate the reliability of their discoveries.

Figure 3, bottom panels, shows that the TPR of all methods increased with the sample size (m) and the number of visits $\left(n_{i}\right)$ , further validating the consistency of all approaches. It is evident that ZINQ-L tCauchy and ZINQ-L MinP demonstrated the highest TPR, with their dominance being more pronounced when the rare taxa or a mixture of common and rare taxa were simulated with signals. This greater power gain arises because the sparsity of rare taxa typically leads to diminished mean differences while pushing signals to the tail, reducing the power of mean-based methods, whereas quantile-based approaches are sensitive to tail events. Among the competing methods, LinDA, particularly when common taxa had signals, provided remarkably low FDR while maintaining an acceptable TPR (Figure 3, bottom panels). Although its TPR was inferior to ZINQ-L, it ranked among the top competitors, similar to ZIGMM.

The results further highlight the advantages of ZINQ-L in realistic settings involving OTU tables and adjusted longitudinal analysis. Its FDR is comparable to existing methods, imposing no additional burden of false discoveries. At the same time, it significantly enhances TPR, primarily by identifying heterogeneous longitudinal associations, such as crossing effects and tail events.

## 6 Application

We applied ZINQ-L to the KTx study to assess the associations between individual taxa and antibiotic treatment over time, adjusting for age and gender. Age was normalized prior to the analysis. Detailed data pre-processing has been described in Section 3. For comparison, we also applied LMM, LinDA, MaAsLin2, and ZIGMM methods, but excluded ZINBMM due to its inflated type I error in Simulation 1. Individual taxa p-values were adjusted using the BH procedure, and taxa with adjusted p-values less than 0.05 were considered differentially abundant over time.

**FIGURE 4:**
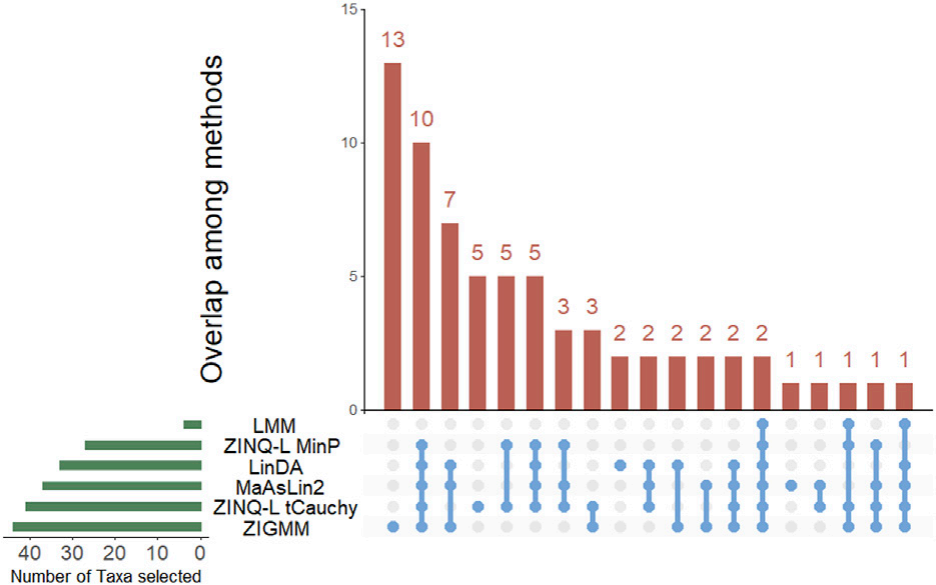
UpSet plot shows the number of taxa identified by each method, and their intersections.

**FIGURE 5:**
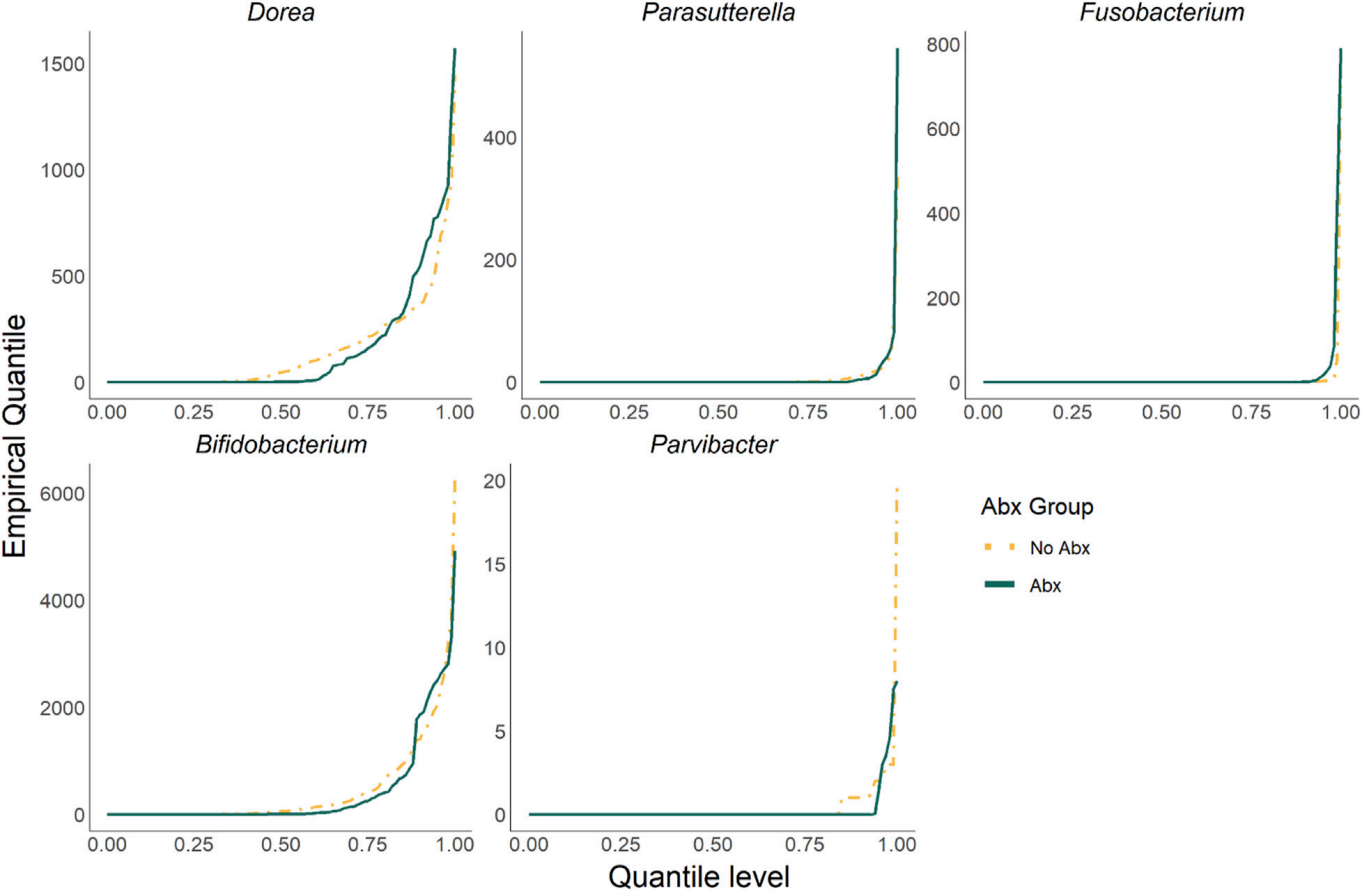
The plot of empirical quantiles (stratified by Abx and No Abx samples) of taxa identified exclusively by ZINQ-L.

Figure 4 shows that a total of five taxa were identified by ZINQ-L tCauchy and ZINQ-L MinP but not by any competing methods. These taxa are *Dorea*, *Parasutterella*, *Fusobacterium*, *Bifidobacterium*, and *Parvibacter*. We plotted their empirical quantiles, stratified by Abx and No Abx samples, in Figure 5. Most of the unique taxa identified by ZINQ-L are rare, consistent with Simulation 2, which shows ZINQ-L’s superiority in identifying tail differences in rare taxa. For example, *Fusobacterium* is enriched by antibiotics at the tail, and while the excessive zeros hinder mean-based detection, ZINQ-L successfully captured it. The relatively common taxa, *Dorea* and *Parvibacter*, show crossing effects: they are enriched by antibiotics when the patients already have abundant *Dorea* and *Parvibacter* in their gut, but depleted by antibiotics when bacterial abundance is low. This finding aligns with Simulation 1, further confirming ZINQ-L’s greater power in identifying crossing effects.

To provide a clearer demonstration, we also created an UpSet plot for ZINQ-L MinP, MaAsLin2 and ZIGMM (Supplementary Figure S2). ZINQ-L MinP identified five taxa that were not detected by MaAsLin2 and ZIGMM. We also plotted the empirical quantiles for two of these uniquely identified taxa: *Enterococcus* and *Eubacterium*. For *Enterococcus*, the Abx and non-Abx groups displayed markedly different proportions of zeros. Furthermore, there was a notable crossing in the empirical quantiles between the groups for *Eubacterium*, indicating significant differences in their distributions.

Importantly, these uniquely identified taxa by ZINQ-L have been reported to be associated with antibiotic usage. For instance, *Dorea* has been increasingly linked to the use of macrolides as noted by (Shah et al., 2021). Conversely, *Bifidobacterium* is reported to be susceptible to penicillin and amoxicillin according to (Delgado et al., 2005). Additionally, *Fusobacterium* has been documented to interact with antibiotics in various conditions, including colorectal cancer (Bullman et al., 2017) and respiratory infections (Li et al., 2020). The established significance of these bacteria in relation to antibiotic treatment highlights ZINQ-L’s value in complementing and improving existing biomarker discovery in longitudinal microbiome studies.

## 7 Discussion

In this paper, we introduce ZINQ-L, a robust and powerful method for identifying associations between individual taxa and a phenotype of interest in longitudinal microbiome studies. ZINQ-L is based on a two-part quantile regression model for longitudinal data. It includes a mixed-effects logistic regression to detect differences in a taxon’s presence-absence status and a series of quantile rank-score-based tests that consider within-subject correlations and zero inflation to detect distributional differences in abundance, given the taxon’s presence. An omnibus p-value, which integrates the marginal tests using the MinP procedure or the truncated Cauchy combination test, indicates whether the distribution of taxon abundance varies with the variable of interest over time. By design, ZINQ-L is a non-parametric regression approach, robust to the complex distributions of microbiome data, and flexible to adjust for covariates. By comprehensively examining the entire abundance distribution, ZINQ-L is also able to identify heterogeneous signals beyond simple mean shifts.

The two options, ZINQ-L MinP and ZINQ-L tCauchy, each have their own advantages and disadvantages. ZINQ-L MinP leverages the dependence structure of the marginal tests, but calculating the omnibus p-value through resampling is time-consuming. Conversely, ZINQ-L tCauchy, which is a weighted sum of multiple transformed p-values, is robust mainly at the tail but not generally (Fang et al., 2023). However, its straightforward calculation ensures computational efficiency. Simulation studies have shown that both options successfully control the FDR below the nominal level when analyzing the OTU table. However, when analyzing individual taxa, ZINQ-L MinP effectively controls Type I error, whereas ZINQ-L tCauchy exhibits slight inflation of Type I error. Despite this, ZINQ-L tCauchy consistently demonstrates greater power than ZINQ-L MinP. This finding is supported by both simulation studies and real data analyses, where the taxa identified by ZINQ-L MinP form a subset of those identified by ZINQ-L tCauchy. Therefore, ZINQ-L MinP is recommended as the default, while ZINQ-L tCauchy is suggested for large-scale analyses or when computational efficiency is critical.

ZINQ-L demonstrates comparable or improved power/TPR compared to existing methods while effectively controlling type I error/FDR. Its enhanced power is particularly evident when heterogeneous associations, such as crossing effects or tail events, are present. This is supported by Simulation 1, which shows ZINQ-L’s superior performance for *Blautia* and *Dorea* with crossing effects, and by Simulation 2, where rare taxa are differentially abundant with predominantly tail events, making ZINQ-L’s improvement more pronounced.

It is important to note that the null hypotheses tested by these methods are not identical. ZINQ-L, like its predecessor ZINQ, assesses differential relative abundance in a general sense, reflecting the compositional nature of the microbiome data. In contrast, LMM, ZIGMM, and ZINBMM are generic longitudinal methods with different distributional assumptions but similarly test for differential relative abundance. MaAsLin2, meanwhile, analyzes log-transformed relative abundances by default. Conversely, LinDA operates under the assumption that the majority of taxa are not differentially abundant; it compares the regression coefficient of each taxon to the mode of all coefficients, thereby targeting differential absolute abundance. These variations in the underlying null hypotheses may partly account for the observed differences in TPR performance among these methods.

When analyzing the KTx study, ZINQ-L identified five unique taxa that the traditional linear mixed model and the tailored methods, LinDA, MaAsLin2, and ZIGMM, failed to capture. Among these taxa, *Fusobacterium* is enriched by antibiotics at the tail, while *Dorea* and *Parvibacter* exhibit crossing effects in response to antibiotic treatment. This observation is consistent with the results from the simulation studies. Biologically, the tail and crossing effects indicate diverse antibiotic effects that depend on the bacteria’s abundance level. Such abundance-dependent effects are crucial for understanding complex pathological mechanisms and devising precision therapeutics. Moreover, the biomedical literature has reported associations of these taxa with antibiotic usage, further validating ZINQ-L’s value for real-world biomarker discovery in longitudinal microbiome studies. Notably, LMM identified only 4 taxa associated with Abx, compared to 33 by LinDA and 37 by MaAsLin2, although none of them specifically adjust for the zero inflation. This discrepancy could stem from LMM’s reliance on the normality assumption for count data, which does not hold in practice due to the high skewness of microbiome data. Conversely, LinDA and MaAsLin2 employ a log transformation on the original counts to better approximate a normal distribution. In contrast, ZINQ-L does not depend on any specific distributional assumptions, potentially enhancing its power to analyze such data.

There are several limitations of ZINQ-L. First, as a non-parametric method requiring a series of estimations across multiple quantiles, ZINQ-L loses power with small sample sizes, particularly when the differences are primarily mean shifts. However, with the increasing availability of large-scale longitudinal microbiome studies, ZINQ-L’s advantage in identifying biologically meaningful heterogeneous effects becomes more significant. Additionally, the current ZINQ-L framework does not accommodate non-linear associations between the quantiles of taxon abundance and covariates. Extending the framework to single-index quantile regression models (Ma and He, 2016) could provide greater flexibility and potentially higher testing power.

## 8 Conclusion

ZINQ-L is a novel approach for examining heterogeneous associations between individual taxa and outcomes (or exposures) over time in longitudinal microbiome studies. It investigates inflated zeros using a logistic mixed-effects model and analyzes the taxon distribution, given its presence, using a marginal quantile mixed-effects model. The marginal tests are then combined using the MinP procedure or the truncated Cauchy test. By design, ZINQ-L effectively handles the complex distribution of microbiome data and within-subject correlations in longitudinal data. Simulation studies demonstrate that, when analyzing individual taxa or the entire OTU table, ZINQ-L controls type I error/FDR and shows improved power/TPR compared to existing approaches. The KTx data analysis further reveals that ZINQ-L can uniquely identify five taxa that have heterogeneous associations with antibiotic usage. These diverse antibiotic effects depending on the bacteria’s abundance level are crucial biomedical findings for uncovering complex pathological mechanisms. Additionally, according to existing literature, these findings are potentially critical for devising antibiotic treatment regimens or understanding the immune system. Overall, ZINQ-L is a robust and powerful tool that complements and enhances current methodsfor identifying associations between individual taxa and the phenotype of interest in longitudinal studies.

## Data Availability

Original datasets are available in a publicly accessible repository: the KTx data, sequencing and de-identified clinical data, presented in this study are deposited in dbGaP with accession number phs001879.v2.p1. Local institutional review board approval will be needed to access the dataset. The R package ZINQ-L is available at https://github.com/AlbertSL98/ZINQ-L in formats appropriate for Macintosh, Windows, or Linux systems.

## Appendix

### Mild conditions for asymptotics of ZINQ-L

Assumption 1. Let F denote the common marginal distribution of $u_{ij}$ for any $i=1,\ldots ,m,\;j=1,\ldots ,n_{i}$ and f denote the Lebesgue density of function F . The joint distribution of $u_{ij_{1}}$ and $u_{ij_{2}}$ for any i and $j_{1}\neq j_{2}$ , denoted by $F_{1,2}$ , is Lipschitz in the neighborhood of (0,0) .

Assumption 2. Let $\left\|s_{ij}\right\|$ denote the Euclidean norm of $s_{ij}$ , where $s_{ij}={\left(x_{ij},z_{ij}^{⊤}\right)}^{⊤}$ . Then $\max_{ij}\|s_{ij}\|=O\left(N^{1/4}\right)$ and $N^{-1}\sum_{ij}\|s_{ij}{\|}^{3}=O\left(1\right)$ as N→∞ .

Assumption 3. The minimum eigenvalues of $E\left(\tilde{X}{\tilde{X}}^{⊤}\right)$ and $E\left(\tilde{Z}{\tilde{Z}}^{⊤}\right)$ are bounded away from 0 as N→∞ .

Assumption 4. The sequence $\left\{n_{i},\;i=1,\ldots ,m\right\}$ is a uniformly bounded sequence of positive integers.

### Computational efficiency

Computations were performed on the Joint High Performance Computing Exchange (JHPCE), maintained by the Department of Biostatistics at the Johns Hopkins Bloomberg School of Public Health. The computing node used is equipped with a 12-core 2.1 GHz Intel Xeon Silver 4310 processor. Table 1 summarizes the total time required to run 10 random simulated OTU tables across various combinations of m and $n_{i}$ , or to run the KTx analysis 10 times. ZINQ-L is less computationally efficient than its competitors, as both the logistic mixed model and the estimation of the rank-score variance $Q_{N}\left(\tau \right)$ under the longitudinal setting are time-consuming. For the two options of ZINQ-L, ZINQ-L tCauchy is faster than ZINQ-L MinP, making it more suitable for large-scale data analysis.
