# Supplementary material for: ZINQ-L: a zero-inflated quantile approach for differential abundance analysis of longitudinal microbiome data
Source: Front Genet. 2025 Jan 29;15:1494401. doi: 10.3389/fgene.2024.1494401 (PMC11814158; doi:10.3389/fgene.2024.1494401)
Supplement: Supplementary file 1 [file DataSheet1.pdf]

Supplementary Information of "ZINQ-L: A Zero-Inflated Quantile Approach for Differential Abundance Analysis of Longitudinal Microbiome Data"

Shuai Li, Runzhe Li, John R. Lee, Ni Zhao, Wodan Ling

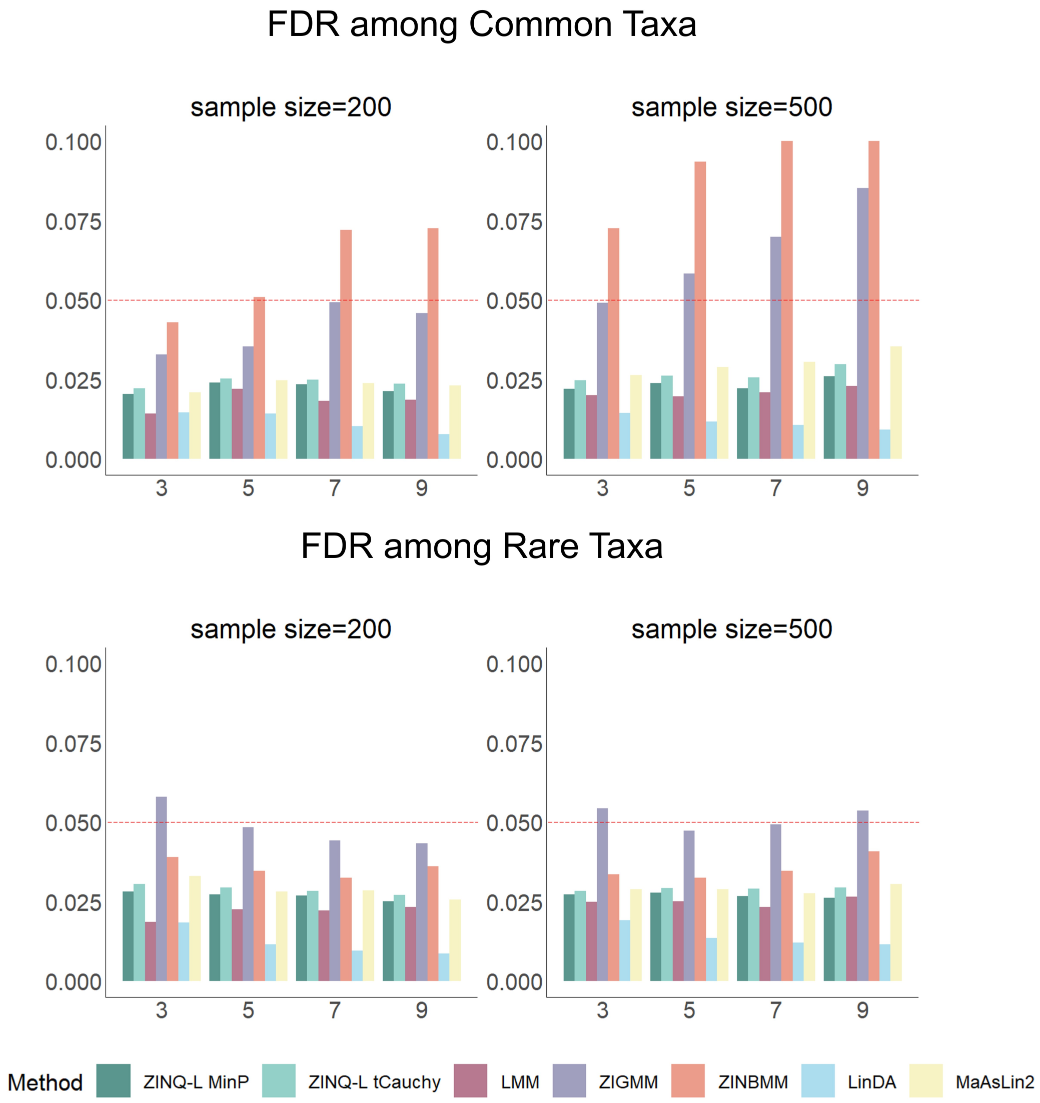

Figure S1: FDR within the common and rare taxa for the simulation scenario when a mixture of common and rare taxa were simulated as differentially abundant. The top panel shows the FDR among the common taxa, which was calculated as $\frac{\text{\#False Discoveries among Common Taxa}}{\text{\#All Discoveries among Common Taxa}}$. The bottom panel shows the FDR among the rare taxa, which was calculated as $\frac{\text{\#False Discoveries among Rare Taxa}}{\text{\#All Discoveries among Rare Taxa}}$.

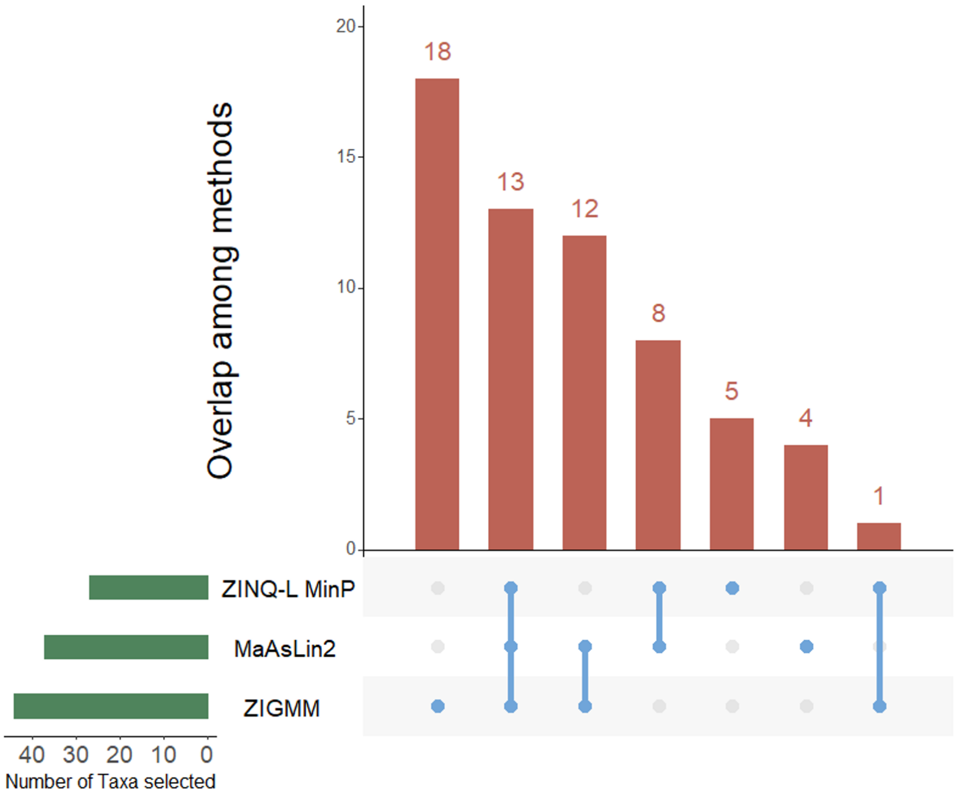

Figure S2: UpSet plot showing the number of taxa associated with Abx identified by ZINQ-L MinP, MaAsLin2 and ZIGMM in the KTx study.

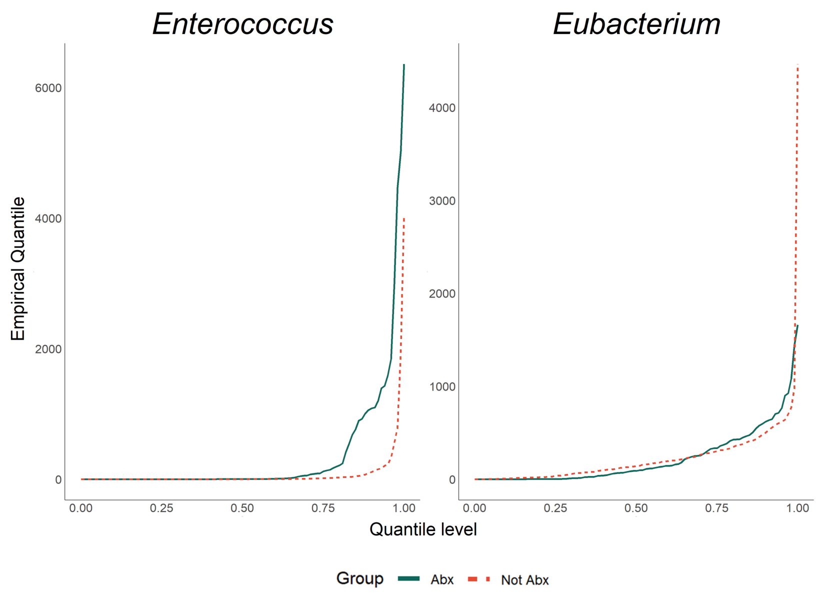

Figure S3: Empirical quantiles for two taxa identified exclusively by ZINQ-L MinP but not MaAsLin2 and ZIGMM in the KTx study.
